# Supplementary material for: Performance estimation of two in-house ELISA assays for COVID-19 surveillance through the combined detection of anti-SARS-CoV-2 IgA, IgM, and IgG immunoglobulin isotypes
Source: PLoS One. 2023 Feb 6;18(2):e0270388. doi: 10.1371/journal.pone.0270388 (PMC9901778; doi:10.1371/journal.pone.0270388)
Supplement: S1 Table — List of all reagents and instruments (brand and source) used to run both in-house ELISAs. (PDF) [file pone.0270388.s001.pdf]

S1 Table. In-house ELISAs reagents and instruments. List of all reagents and instruments (brand and source) used to run both in-house ELISAs.

REAGENTS

Np protein: Recombinant SARS-CoV-2 Np protein (R&D System, Catalog Number 10474.CV) (accession # YP_009724397.2). (Minnesota, USA)

RBD protein: Recombinant SARS-CoV-2 S1 subunit protein (Raybiotech, Catalog Number: 230-30162) (accession # QHD43416.1). (Georgia, USA)

96-well plate: Corning 96 well flat clear bottom white (Corning Life Science). (Massachusetts, USA).

PBS: Phosphate Buffer Saline (Winkler Ltda, Chile)

Tween20: (Santa Cruz Biotechnology, Inc.) SC29113 (California, USA)

BSA: Bovine serum albumin - Fraction V (Immunoglobulin and Protease Free) Rockland Immunochemicals, Inc. (Pensilvania, USA)

Anti-Human IgA/IgG/IgM (H&L) goat polyclonal antibody (HRP) (Rockland, R.609-103-130)

TMB: Substrate (3, 3', 5, 5' – Tetramethylbenzidine (Santa Cruz Biotechnology, Inc.) (California, USA)

LFCI kit: The Diagnostic Kit for IgM / IgG Antibody to Coronavirus (SARS-CoV-2) (Lateral Flow), LIVZON, China).

INSTRUMENTS:

Portable centrifuge: (Mobilspine, Vulcon Technologies, Richmond, USA)

Ultrafreezer -80°C: Thermo Forma 700 Series (Marietta, USA)

fridge at 4°C

Microplate Reader (96 well): HR801, Shenzhen Highcreation Technology Co. Ltd. (China).

Incubator (37° C): BM 400, Memmert (Switzerland, Aargau)

Refrigerator (Cooler at 4°C): Mabe (Ciudad de México, México),
