# Supplementary material for: Performance estimation of two in-house ELISA assays for COVID-19 surveillance through the combined detection of anti-SARS-CoV-2 IgA, IgM, and IgG immunoglobulin isotypes
Source: PLoS One. 2023 Feb 6;18(2):e0270388. doi: 10.1371/journal.pone.0270388 (PMC9901778; doi:10.1371/journal.pone.0270388)
Supplement: S2 Table — Bulk cost of all reagents used to run both in-house ELISAs. Figures have been converted from Chilean Pesos (CLP) to US dollar (USD) using exchange rate of July 2020, that correspond to the date when regents were bought. (PDF) [file pone.0270388.s002.pdf]

S2 Table. Cost of in-house ELISA reagents. Bulk cost of all reagents used to run both in-house ELISAs. Figures have been converted from Chilean Pesos (CLP) to US dollar (USD) using exchange rate of July 2020, that correspond to the date when regents were bought.

ELISA Np for antibody detection for SARS-CoV2

SPECIFIC MATERIALS (MATRIX-PEPTIDE-ANTBODIES)	SUPPLIER	CODE	VALUE (CLP)	Unit	TOTAL
Recombinan SARS-CoV 2, Np (HIS-Tagged) 50 ug	RAYBIOTECH-GENEXPRESS	RD.10474-CV-050	361,000	1	361,000
Anti Human IgG, IgA e IgM (H&L) antibody HRP	ROCKLAND-GENEXPRESS	R.609-103-130	289,180	1	289,180
**REAGENTS**					
BSA FRACCIONV	ROCKLAND-GENEXPRESS	RBSA-50	138,915	3	416,745
TWEEN 20 (100ml)	SANTACRUZ-FERMELO	SC29113CSANTA	9,900	1	9,900
TBST(10X)	ALLSHENG-WINKLER	719520	49,200	4	196,800
PBS(10X)	ALLSHENG-WINKLER	713420	44,700	4	178,800
PAPEL 150 MT (PARA DISPENSADOR)			9,000	6	54,000
**PLASTICS**					
TUBO 50 ML (500U)	ALLSHENG-WINKLER		58,000	1	58,000
TUBO 15ML (50U)	SANTACRUZ-FERMELO		4,600	5	23,000
TUBO 2,0 ML (500U)	ALLSHENG-WINKLER		13,000	6	78,000
TUBO 1,5ML (500u)	GENEXPRESS		10,164	6	60,984
PUNTAS BLANCAS(P10)	GENEXPRESS		10,438	10	104,380
PUNTAS AMARILLA(P100)	GENEXPRESS		5,990	10	59,900
PUNTAS AZULES (P1000)	GENEXPRESS		10,675	10	106,750
PLACA96 POSILLOS (25u)	GENEXPRESS		22,760	5	113,800
PARAFILM	GENEXPRESS	BEM.PM99D	18,404	1	18,404

**TOTAL NETO**		**2,129,643**
**IVA (19%)**		**1,622,101**
**TOTAL**		**3,751,744**

1250 samples in duplicate based on Np	2500 analyses in total	1200 samples		3,126
		USD/CLP rate	739	
		**Cost (USD)per sample**		**4.23**

ELISA RBD for antibody detection for SARS-CoV2

SPECIFIC MATERIALS (MATRIX-PEPTIDE-ANTBODIES)	SUPPLIER	CODE	VALUE (CLP)	Unit	TOTAL
Recombinan SARS-CoV 2, S1 (HIS-Tagged) 100ug	RAYBIOTECH - GENEXPRESS	RB23030162-100 UG	379,003	1	379,003
Anti Human IgG, IgA e IgM (H&L) antibody HRP	ROCKLAND-GENEXPRESS	R.609-103-130	289,180	1	289,180
**REACTIVOS**					
BSA FRACCIONV	ROCKLAND-GENEXPRESS	RBSA-50	138,915	1	138,915
TWEEN 20 (100ml)	SANTACRUZ-FERMELO	SC29113CSANTA	9,900	1	9,900
TBST(10X)	ALLSHENG-WINKLER	719520	49,200	1	49,200
PBS(10X)	ALLSHENG-WINKLER	713420	44,700	1	44,700
PAPEL 150 MT (PARA DISPENSADOR)			9,000	4	36,000
**PLASTICOS**					
TUBO 50 ML (500U)	ALLSHENG-WINKLER		58,000	1	58,000
TUBO 15ML (50U)	SANTACRUZ-FERMELO		4,600	4	18,400
TUBO 2,0 ML (500U)	ALLSHENG-WINKLER		13,000	4	52,000
TUBO 1,5ML (500u)	GENEXPRESS		10,164	4	40,656
TUBO CRIOGENICO 2ML (50u)	GENEXPRESS		8,751	4	35,004
PUNTAS BLANCAS(P10)	GENEXPRESS		10,438	4	41,752
PUNTAS AMARILLA(P100)	GENEXPRESS		5,990	4	23,960
PUNTAS AZULES (P1000)	GENEXPRESS		10,675	4	42,700
PLACA96 POSILLOS (25u)	GENEXPRESS		22,760	2	45,520
PARAFILM	GENEXPRESS	BEM.PM99D	18,404	1	18,404
**EQUIPO/DISPOSITIVOS**					
SET MICROPIPETAS LAMBDAPLUS STARTER KIT (P10-P20-P200-P1000-SOPORTE)	GENEXPRESS	CLS.4069	520,000	1	520,000
PIPETA MULTICANAL (8) SILOGEX	SILOGEX-WINKLER	WS6000	279,000	1	279,000

**TOTAL NETO**		**2,122,294**
**IVA (19%)**		**1,622,101**
**TOTAL**		**3,744,395**

1958 samples in duplicate based on RBD	3916 analyses in total	1200 samples		1,958
		USD/CLP rate	739	

**Cost (USD)per sample**	**2.65**
